# Atrial and ventricular kinetic energy is higher in athletes compared to healthy controls and contributes to improve diastolic filling of the ventricles

**DOI:** 10.1186/1532-429X-17-S1-P30

**Published:** 2015-02-03

**Authors:** Katarina Steding-Ehrenborg, Per M Arvidsson, Mattias Rydberg, Marcus Carlsson, Håkan Arheden

**Affiliations:** 1 Cardiac MR group Lund, Dept. of Clinical Physiology, Lund University, Lund, Sweden

## Background

The athlete's heart is physiologically enlarged with an extraordinary ability to pump large volumes of blood at high heart rates. We hypothesized that efficient filling and emptying is related to the kinetic energy (KE) of intra-cardiac blood. Therefore, the aim of this study was to investigate variations in KE in the four chambers of the heart over the cardiac cycle and compare endurance athletes to healthy controls.

## Methods

Fourteen athletes (8 women) and 14 controls (6 women) underwent CMR with a 5-channel cardiac coil in a 1.5T Philips Achieva or 3T Philips Intera (Best, The Netherlands). Three-dimensional time-resolved phase contrast flow (4D PC) was performed using a box covering the whole heart. Typical imaging parameters were: TE/TR 3/5ms, α 8°, SENSE factor 2, velocity encoding 100cm/s, spatial resolution 3x3x3mm, acquired temporal resolution of 40ms with a reconstructed temporal resolution of 25ms. Kinetic energy was calculated as KE=0.5*mv*^2^ and rotational KE of the atria was calculated as KE=0.5*mv_α_*^2^ where *m* is mass, *v* is velocity and *v_α_*is the angular velocity about the rotational axis.

## Results

**Figure 1:**
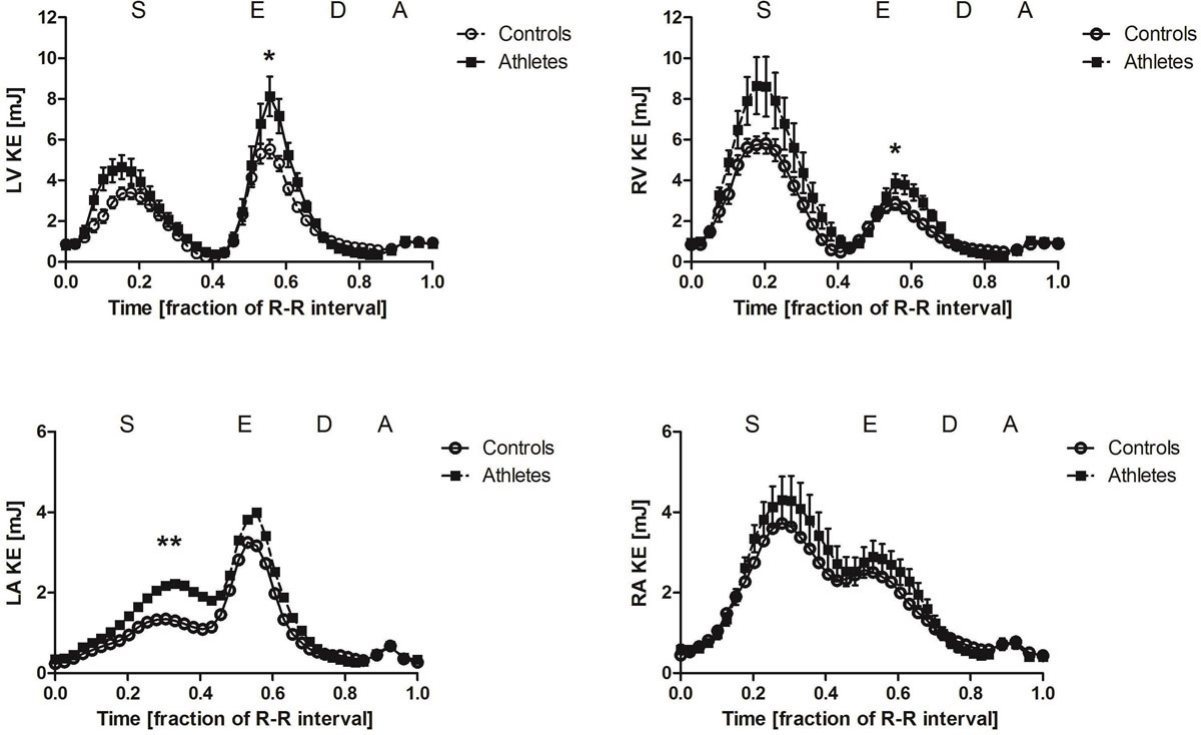
Kinetic energy in the ventricles and atria over the cardiac cycle in athletes and controls (mean±SEM). Upper panel: Left ventricular kinetic energy (LV KE) and right ventricular kinetic energy (RV KE) was higher during early diastole in athletes compared to controls. Lower left: Left atrial kinetic energy (LA KE) was higher during systole in athletes compared to controls. Lower right: Right atrial kinetic energy (RA KE) did not differ between groups. *S - systole*, *E - early diastole*, *D - diastasis*, *A - atrial contraction*, *mJ - milli-joule*, *p<0-05. **p<*0.01*

**Figure 2:**
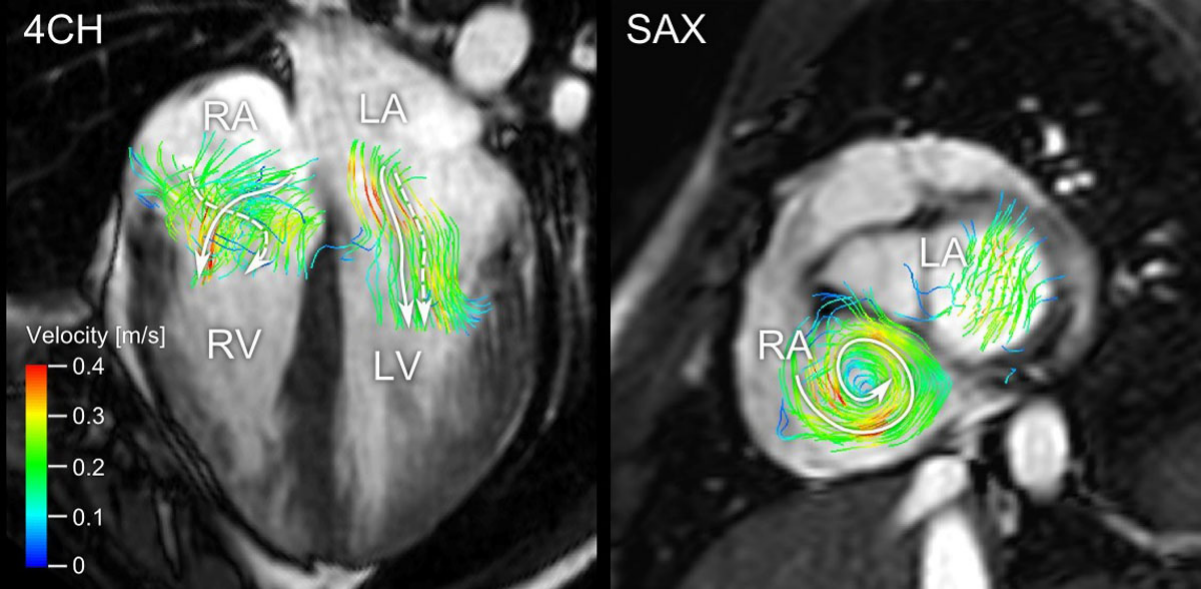
Visualization of rotational kinetic energy in the left and right atrium of an elite endurance athlete. Left panel shows left and right atrial flow in a 4 chamber image and right panel shows the same flow in a short-axis image. Note the helical flow from the right atrium into the right ventricle. *LA - left atrium*, *RA - right atrium*, *LV - left ventricle*, *RV - right ventricle*, *4ch - 4 chamber view*, *SAX - short-axis view.*

Three energy peaks were seen in all 4 chambers in both groups; during systole, early diastole (E-wave) and late diastole (A-wave) (Figure 1). Athletes had higher energy peaks compared to controls in early diastole for the left and right ventricle (8.87±1.06mJ vs 5.89±0.44 for LV and 4.24±0.49 vs 3.09±0.36 for RV, p<0.05) and during ventricular systole in the left atria (2.43±0.25mJ vs 1.44±0.15, p<0.01). There was a correlation between left ventricular mass (LVM) and peak KE during early diastole of the left ventricle, indicating a greater diastolic suction in subjects with large LVM (R^2^=0.66, p<0.001). Rotational KE in the left and right atria did not differ between groups. During the early diastolic peak in the left atrium the average angle between net blood flow direction and axis of rotation was 81° for athletes and 91° for controls indicating mainly non-helical flow. Conversely for the right atrium, the angle was 35° for athletes and 37° for controls, indicating more helical flow from the right atrium into the ventricle (Figure 2).

## Conclusions

Kinetic energy varies over the cardiac cycle in a similar way in athletes and controls although athletes have higher peak values. A large LVM was correlated to a high early diastolic peak of the left ventricle, indicating an improved diastolic suction in athletes at rest that may become even more significant during exercise.

## Funding

This study was funded by World Village of Women Sports and the Swedish Centre for Sports Science.

